# Evidence for use of both capital and income breeding strategies in the mangrove tree crab, *Aratus pisonii*

**DOI:** 10.1038/s41598-021-94008-8

**Published:** 2021-07-16

**Authors:** Jade Carver, Morgan Meidell, Zachary J. Cannizzo, Blaine D. Griffen

**Affiliations:** 1 Biology Department, Brigham Young University, Provo, UT 84602 USA; 2 National Oceanic and Atmospheric Administration Office of National Marine Sanctuaries – National Marine Protected Areas Center, Silver Spring, MD 20910 USA

**Keywords:** Ecology, Physiology, Ecology

## Abstract

Two common strategies organisms use to finance reproduction are capital breeding (using energy stored prior to reproduction) and income breeding (using energy gathered during the reproductive period). Understanding which of these two strategies a species uses can help in predicting its population dynamics and how it will respond to environmental change. Brachyuran crabs have historically been considered capital breeders as a group, but recent evidence has challenged this assumption. Here, we focus on the mangrove tree crab, *Aratus pisonii*, and examine its breeding strategy on the Atlantic Florida coast. We collected crabs during and after their breeding season (March–October) and dissected them to discern how energy was stored and utilized for reproduction. We found patterns of reproduction and energy storage that are consistent with both the use of stored energy (capital) and energy acquired (income) during the breeding season. We also found that energy acquisition and storage patterns that supported reproduction were influenced by unequal tidal patterns associated with the syzygy tide inequality cycle. Contrary to previous assumptions for crabs, we suggest that species of crab that produce multiple clutches of eggs during long breeding seasons (many tropical and subtropical species) may commonly use income breeding strategies.

## Introduction

Reproduction is energetically expensive and therefore requires that organisms develop life history strategies geared towards financing this crucial biological process^1^. The strategies for energy storage and allocation that are used to meet the costs of reproduction can vary widely across species^2^. Two broad strategies, capital and income breeding, are distinguished by the timing of resource investment in reproductive efforts versus somatic buildup in relation to the breeding season^2^. These two reproductive strategies are delineated by the use of energy for reproduction that is either stored prior to reproduction (capital breeding) or obtained during the reproductive period (income breeding)^3^. While organisms could potentially be relatively strict capital or income breeders, oftentimes a blend between strategies is used^4,5^. Therefore, the distinction between capital and income breeding can rather be considered a continuum of investment strategies, from all reproduction being financed by current energy intake to all reproduction being financed by energy reserves^3^.

Understanding where species lie on the capital-to-income breeding continuum, can help in discerning the causes of population cycles and outbreaks at irregular intervals^6^, predicting how they will react in the face of environmental changes (both natural and/or human-induced), or anticipating invasion success as they enter new environments^7^. For instance, it is likely that the demographics of capital breeders are more affected by shifts in environmental condition outside of their breeding season, while income breeders may be more affected by environmental conditions within the breeding season^2^. Additionally, organisms capable of plasticity in resource allocation, allowing them to shift along the capital-to-income breeding continuum, may experience greater resilience to environmental change^8^. These predictions have been supported in a number of organisms. For instance, caribou and muskoxen occur in the same environment and commonly use the same resources, but caribou reproduce primarily using an income strategy, while muskoxen generally use a capital breeding strategy. During years with poor resource supply during the breeding season, caribou reproduction decreases, while muskoxen reproduction is unaffected; however, muskoxen are sensitive to poor resource conditions from earlier years^9^.

Energetically financing reproduction is central to all types of organisms, and brachyuran crabs have recently been the focus of studies examining breeding strategies within crustaceans^10,11^. Crabs play prominent ecological^12^ and economic roles^13^ in coastal systems, and understanding the diversity of reproductive strategies within brachyuran crabs could help in predicting how this group will respond to climate change or other human-induced environmental changes that alter the timing of food availability^14^. Typically, it has been assumed that crabs and other small ectotherms are primarily capital breeders due to the relatively low cost of energy storage they experience^3^. In crabs, this assumption is supported by previous studies that document an inverse relationship between hepatopancreas (a digestive organ) and ovary mass^15,16^. Specifically, decreasing lipid stores in the hepatopancreas is often accompanied by an increase in ovary mass, suggesting the transfer of stored energy for the process of vitellogenesis^11^. However, recent studies have shown that this is not always the case, as some crab species (i.e., the European green crab *Carcinus maenas* and the blue crab *Callinectes sapidus*) employ income breeding or possibly use a combination of both breeding strategies^11,17^. Additionally, theoretical work suggests that the costs of energy storage in small-bodied ectotherms may not be as low as previously assumed^18^. Thus, the extent to which crabs use capital vs. income breeding strategies remains unclear.

The use of capital vs. income breeding strategies may also be influenced by the degree of seasonality and the number of reproduction events per year. For temperate organisms that have an active feeding and a clear breeding season with multiple clutches of eggs produced per season, a proposed strategy includes using capital breeding at the start of the reproductive period to fund the first few clutches of eggs, then turning to income breeding for the remainder of reproductive events, once capital has been expended^19^. This blended strategy is used by other crustaceans, including the copepod *Calanoides acutus*^19,20^. Such a combination of strategies allows early clutches to be comprised of mostly high-quality eggs that can hatch and develop during the prime feeding time, allowing the offspring to grow and store their own energy prior to the onset of less favorable conditions. Although eggs produced later in the season under income breeding may not have as much time to develop and obtain energy during the season of peak productivity, these additional clutches still have the capacity to increase the fitness of the parents if some of the offspring survive^19,20^.

The mangrove tree crab *Aratus pisonii* provides an opportunity to examine whether single crab species may use both capital and income breeding strategies. A previous attempt to examine breeding strategies across brachyuran crabs returned inconclusive results for *A. pisonii*^11^. This species inhabits a large range throughout the tropics, where it has been documented reproducing continuously year-round in some places^21,22^, while showing seasonal reproductive cycles in others^23^. Females in tropical mangroves mature at 12 mm carapace width and reproduce throughout the year, with individual crabs producing a clutch of eggs approximately every 66 days^21^. At the extremes of its range on the Atlantic coast of Florida, *A. pisonii* experiences seasonality and produces multiple egg clutches during a defined reproductive season, facilitating the inspection of energy allocation before and during the breeding season^24^. A previous experiment conducted during the early part of the reproductive season found that energy allocated to reproduction did not change with the amount of food consumed^25^, suggesting that this species uses capital breeding at the start of its reproductive season, but evidence of income breeding in this species has not been found^11^. Because *A. pisonii* produces multiple egg clutches within a reproductive period, we hypothesize that in Florida, this species uses a combination of capital and income breeding, with the first few clutches funded by stored capital and the later clutches funded by energy obtained during the breeding season^11,20^.

*Aratus pisonii* is primarily herbivorous^26^, but it prefers animal tissue^27^ and includes an appreciable amount of animal tissue in its diet during parts of the year^26,28^. Animal tissue represents a higher quality diet than mangrove leaves, as food assimilation efficiency, survival, energy storage, and reproductive effort all increase as animal tissue increases in the diet of *A. pisonii*^25^. *A. pisonii* has expanded its diet and habitat use^29^, altered its behavioral strategies^30^, and shifted its reproductive patterns in response to climate change^24,31^. Determining its reproductive strategies can therefore aid in anticipating changes in population structure following these shifts and in the face of continued environmental change^32^.

If *A. pisonii* reproduction relies solely or primarily on capital energy stores, then initial energy stores at the start of the reproductive season must meet or exceed the energy invested in egg development throughout the season. Additionally, if stored energy becomes limiting, then reproductive effort should decrease as the reproductive season progresses and stored capital becomes exhausted. Exhausted energy stores may be expressed as smaller clutches (fewer eggs) or reduced per-propagule investment (smaller individual egg masses)^33,34^ as the reproductive season progresses. Alternatively, if reproduction relies substantially on income breeding, then a clear shift in reproductive effort or energy storage is not expected over the duration of the reproductive period. Further, the reliance on an income breeding strategy should necessitate increased energy intake, either via increased amount or quality of food consumed, during periods of peak reproductive output.

We explored these patterns by collecting and dissecting *A. pisonii* from the Florida coast throughout the reproductive season and measuring reproductive state, the mass of the hepatopancreas and the ovaries, the number and size of eggs, and the amount and quality of dietary intake. We used these data to explore these expected patterns by testing several specific hypotheses, listed in Table 1.

**Table 1:** Hypotheses tested to distinguish between capital and income breeding strategies.

Metric	Hypothesized pattern with capital breeding	Hypothesized pattern with income breeding
(1) Stored energy relative to that required to finance reproduction	Combined energy of hepatopancreas + ovary ≥ cumulative egg energy	Combined energy < cumulative egg energy
(2) Hepatopancreas energy throughout reproductive period	↓ through time as energy stores become depleted	Constant because is not used to finance reproduction
(3) Egg number or size throughout reproductive period	↓ through time as energy stores become depleted	May be variable, depending on dietary energy intake
(4) Diet energy intake throughout reproductive period	Constant, or at least unrelated to reproduction	↑ and ↓ proportional to reproduction

## Results

### Establishing the reproductive season

**Figure 1:**
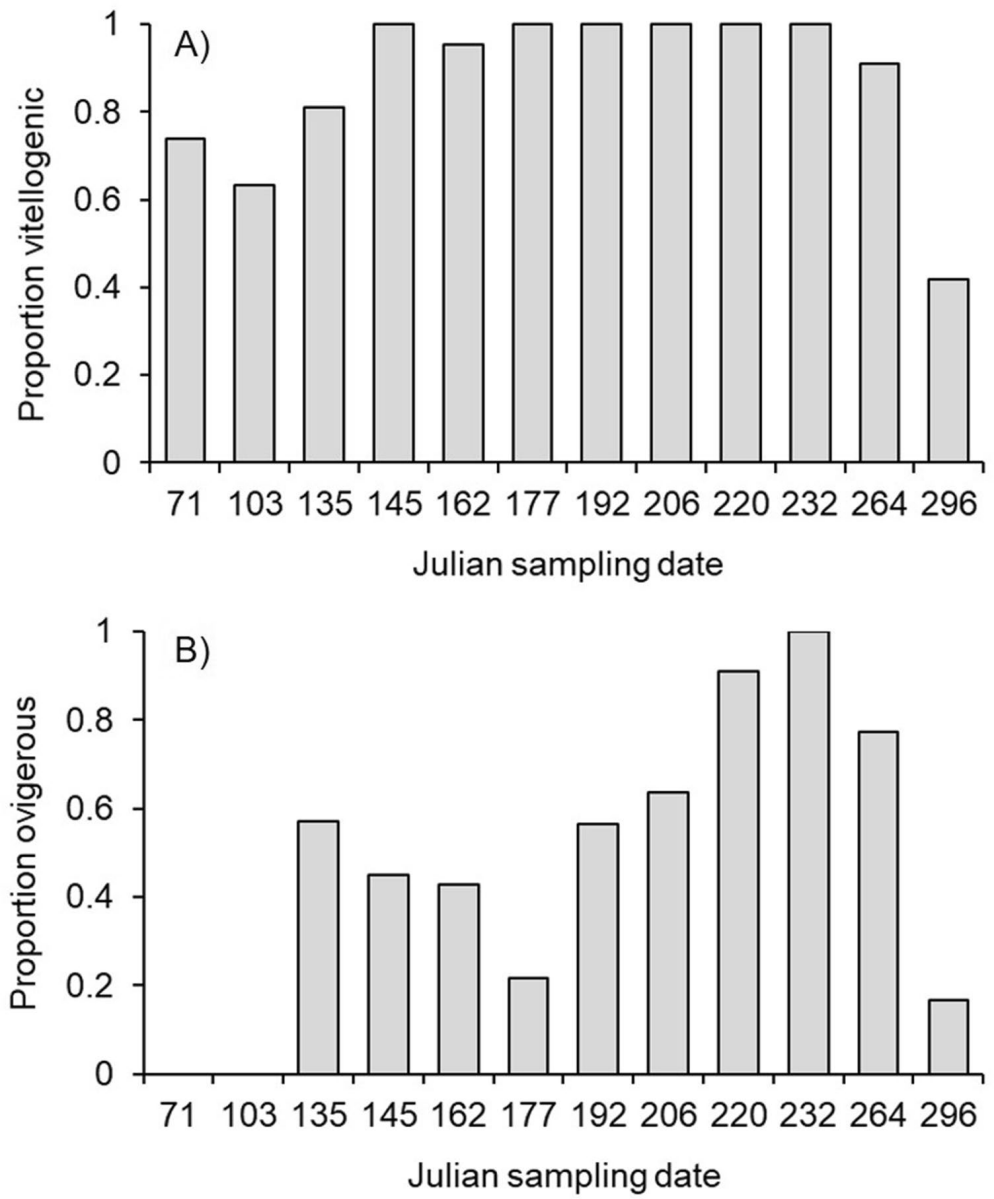
Proportion of *Aratus pisonii* that were vitellogenic (**A**) and ovigerous (**B**) on each Julian sampling date, showing a unimodal distribution consistent with a defined reproductive period.

The probability of being vitellogenic or ovigerous both varied unimodally (i.e., hump-shaped) throughout the sampling period. Vitellogenic crabs comprised 74%, 63%, and 81% of the samples in March (Julian day 71), April (Julian day 103), and May (Julian day 135 and 145), respectively, 98% of the samples from June–September (Julian days 162–264), and dropped to 42% in October (Julian day 296) (binomial GLM, first order polynomial term for Julian date: *z* = 4.83, *P* < 0.0001; second order polynomial term for Julian date: *z* = − 4.69, *P* < 0.0001, Fig. 1A). The probability of being vitellogenic also increased with crab CW (binomial GLM, *z* = 3.77, *P* = 0.0002) and was higher at full moon than at new moon (*z* = 2.23, *P* = 0.026). No ovigerous crabs were encountered in March or April, and only 3 were encountered in October. From May–September, 62% of crabs were ovigerous (binomial GLM, first order polynomial term for Julian date: *z* = 5.67, *P* < 0.0001; second order polynomial term for Julian date: *z* = − 5.45, *P* < 0.0001, Fig. 1B). No other terms were included in the best-fitting model for ovigerous crabs.

### Is stored energy sufficient to finance reproduction?

It does not appear that capital breeding can fully account for even the first clutch of eggs, as the combined mass of the hepatopancreas and the ovaries prior to the appearance of the first ovigerous crabs (i.e., in March and April) was 2.74 × less than the average mass of the first clutch of eggs in May (*t* = 10.32, *P* < 0.0001). This difference was not due to differences in crab size, as no difference existed in CW of crabs sampled before the reproductive season in March and April (17.8 ± 2.8 mm) and those sampled in May (18.6 ± 2.3 mm) (*t* = 1.42, *P* = 0.16).

### Energy storage

**Figure 2:**
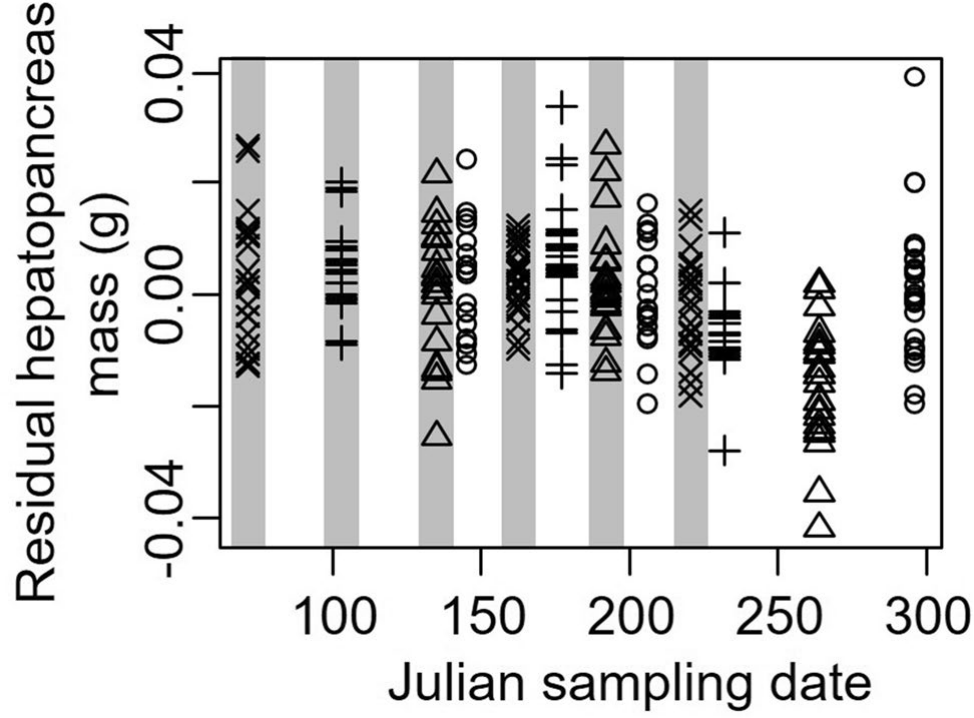
Residual hepatopancreas mass of *Aratus pisonii* (after accounting for body mass) as a function of Julian sampling date. Symbols indicate sampling site, where circles are Round Island, triangles are Oslo, plus sign is North Causeway, and the multiplication sign is Pepper park. Gray shaded sampling dates indicate periods of new moon, while white indicates full moon.

We found that residual hepatopancreas mass (after accounting for body mass) decreased during the sampling period (*t* = − 2.40, *P* = 0.016), consistent with capital breeding, and was an average of 7.82 ± 2.35 mg higher during new moon periods than during full moon periods (*t* = 3.33, *P* = 0.001, Fig. 2). The variance in residual hepatopancreas mass across sites (random effect) was 3.14e-5 ± 0.006, with residual variance 1.11e−4 ± 0.011.

### Egg mass and number

**Figure 3:**
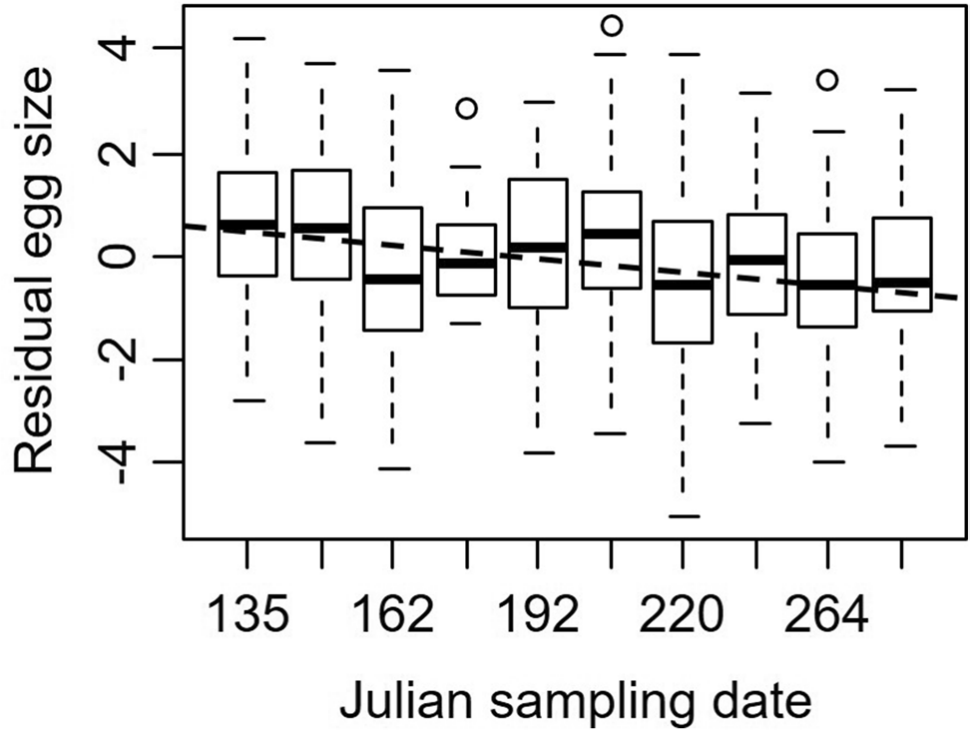
Residual mass of individual *Aratus pisonii* eggs (after accounting for body size) as a function of Julian collection date. The heavy lines of each box plot shows the median, the box encompasses the first and third quartiles of the data, whiskers encompass 95% of the data, and circles indicate outliers that fall outside of this range. The dashed line is drawn to highlight decreasing egg mass through time.

We measured the mass of individual eggs from 123 ovigerous crabs, and the variance in egg mass explained by the random effects of differences between crabs was 0.90 ± 0.95, nested within site 0.06 ± 0.24, with residual variance of 1.38 ± 1.17. The mass of individual eggs decreased by 0.006 ± 0.002 μg with each passing day of the reproductive season (*t* = − 2.96, *P* = 0.004, Fig. 3). Moon phase was not included in the best-fitting model.

**Figure 4:**
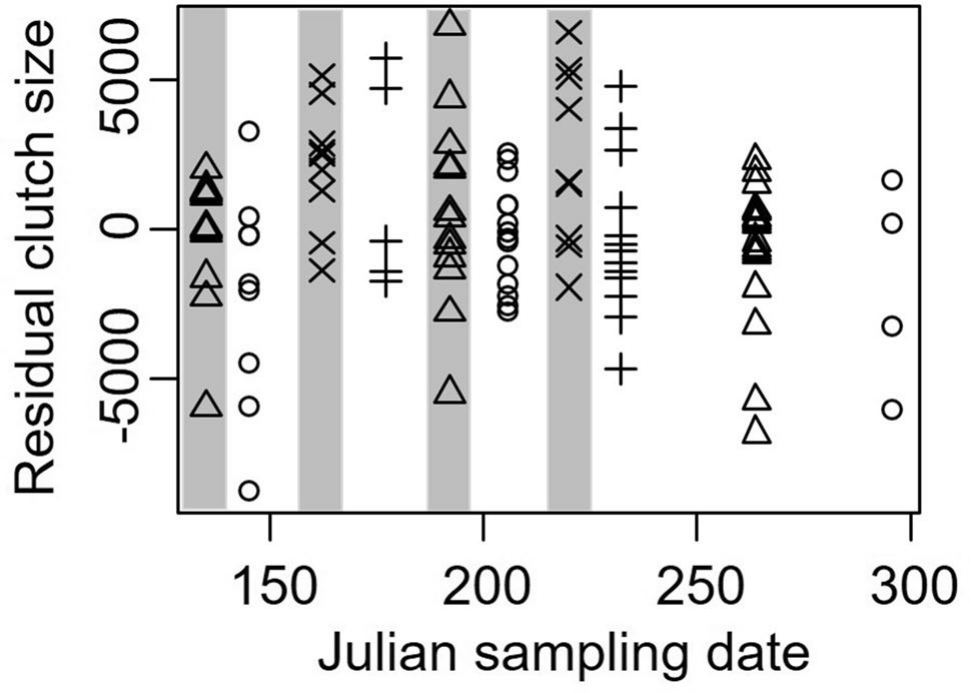
Residual number of eggs per clutch in *Aratus pisonii* (after accounting for body size) as a function of Julian sampling date. Symbols indicate sampling site, where circles are Round Island, triangles are Oslo, plus sign is North Causeway, and the multiplication sign is Pepper park. Gray shaded sampling dates indicate periods of new moon, while white indicates full moon.

In contrast to the size of eggs, the number of eggs in individual clutches did not differ across sampling dates (*t* = 0.83, *P* = 0.41), but was 1,847 ± 838 egg higher during new moon periods than during full moon periods (*t* = 2.20, *P* = 0.05, Fig. 4). The variance in egg number across sites (random effect) was 539,724 ± 735, with residual variance of 7,359,747 ± 2713.

### Diet quantity and quality

**Figure 5:**
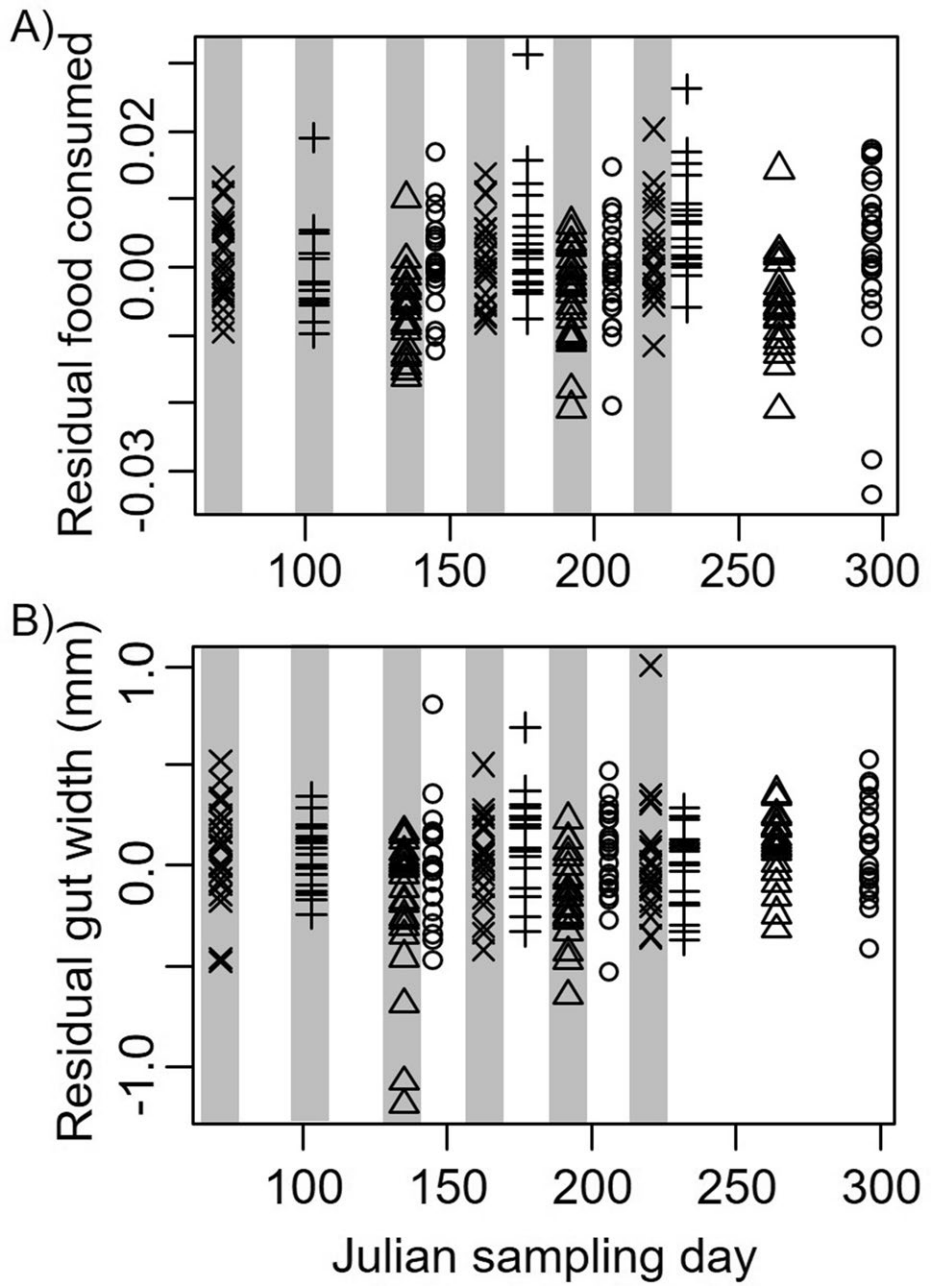
Residual gut mass (**A**) and gut width (**B**) in *Aratus pisonii* (after accounting for body size) as a function of Julian sampling date, proxies for the amount and quality of food consumed, respectively. Symbols indicate sampling site, where circles are Round Island, triangles are Oslo, plus sign is North Causeway, and the multiplication sign is Pepper park. Gray shaded sampling dates indicate periods of new moon, while white indicates full moon.

We found that the residual gut mass (after accounting for body mass) was only influenced by sampling date. Specifically, consistent with income breeding, we found a weak 0.017 ± 0.008 mg increase in gut mass for each day during the sampling period (*t* = 1.98, *P* = 0.05, Fig. 5A). Variance in mass across sites (random effect) was 1.43e−5 ± 0.004, with residual mass of 6e−5 ± 0.007. Diet quality, as determined by the residual gut width after accounting for crab size, was larger for crabs with more food in their guts (*t* = 3.02, *P* = 0.003), justifying our controlling for this factor by including in it our analysis. Overall, we found that residual gut width decreased by 0.1 ± 0.04 mm, indicating higher quality food consumption, during new moon periods compared to full moon periods (*t* = − 2.34, *P* = 0.03, Fig. 5B). Julian sampling day was not included in the best fitting model. Variance in gut width across collecting sites (random effect) was 0.002 ± 0.05, with residual variance of 0.062 ± 0.250.

## Discussion

We have shown that there is a defined breeding season for *A. pisonii* on the Florida coast, and that there is variation in energy intake, energy storage, and energy allocation to reproduction throughout this breeding season that is consistent with the use of both capital and income breeding strategies. Further, the patterns documented here appear to be similar to patterns outlined by^19^ for temperate copepods. Our results also provide new insights into variation in food intake to support temporal patterns of egg production associated with the lunar cycle.

### Evidence for use of both capital and income breeding in this system

We have given two pieces of evidence that clearly support the use of capital breeding in *A. pisonii*. First, we found a decrease in hepatopancreas mass throughout the reproductive period which indicates the gradual depletion of stored energy. Second, we demonstrate that egg mass decreased modestly over the course of the reproductive period, also suggesting that energy stores were becoming depleted and that egg quality consequently declined over time, at least in terms of lipid amount. However, it is possible that egg mass decreases during the reproductive season for reasons other than depletion of capital, such as selection for larger offspring early in the season.

We similarly report two pieces of evidence that support the use of income breeding in *A. pisonii*. First, and most convincingly, we have shown that the average mass stored prior to reproduction in the hepatopancreas and ovaries combined, was 2.74 × less than the average mass of the first clutch of eggs of the reproductive season, meaning that energy from stored capital alone cannot even produce a single clutch, much less all clutches throughout the season. Second, we found that diet quality increased weakly throughout the reproductive season, and that diet quality was higher (i.e., gut sizes were smaller) during new moon periods when clutch sizes were larger than during full moon periods when clutch sizes were smaller. Based on this pattern alone, it is not possible to determine cause and effect. We therefore cannot conclude whether a higher quality diet drove increased reproduction during new moons, or whether higher reproduction at new moon periods stimulated crabs to choose higher quality diets. But either way, the correlation between egg output and diet quality is consistent with income breeding by *A. pisonii*. Results here therefore are consistent with *A. pisonii* supporting reproduction with the use of both stored energy capital and the use of energy brought in throughout the reproductive season. This supports recent work highlighting the use of income breeding in brachyuran crabs that were previously considered to be exclusively capital breeders^11^.

The patterns described above assume that all reproductive females are actively breeding throughout the reproductive time of year. However, *A. pisonii* in São Paulo, Brazil that have a similar seasonal reproductive period^23^, initiate reproduction in two phases, with older individuals initiating reproduction in the spring, and younger individuals reproducing for the first time initiating reproduction in the summer^35^. It is possible that some of the observed patterns could have been influenced by differences in the age of reproducing individuals (i.e., by primarily older individuals reproducing during the early reproductive season and a mix of older and younger individuals reproducing later in the season). However, it is unlikely that age differences could account for all of these patterns that suggest that *A. pisonii* uses a mixture of capital and income breeding strategies. Further clarification of the role of crab age would require longitudinal studies of individual animals throughout the reproductive season.

### Physiological/behavioral correlates to the lunar cycle

As explained in the Methods, our study design included sampling at both new moon and full moon periods. The choice to sample in concert with lunar cycles was methodological, to ensure the capture of crabs during peak reproductive effort. Intertidal crab reproductive cycles are often tied to differences in tidal heights that accompany these lunar cycles^36,37^, and we also found this to be the case with *A. pisonii*. Specifically, we found that reproductive effort (gonad mass and clutch size) was higher during new moon, the period of highest tides during the majority of our sampling period due to the syzygy tide inequality cycle^38^, compared to full moon.

In addition to tidally influenced reproductive patterns that are known to occur in crabs, our data allowed us to explore changes in food intake surrounding the syzygy tide inequality cycle, presumably in support of the cyclical changes in reproduction. Specifically, we found that diet quality and energy storage were both higher during new moon compared to full moon. Previous work with *A. pisonii* demonstrates that diet quality determines fatty acid composition of lipids deposited into eggs, and that this in turn leads to increased survival and rigor for larvae whose mothers consumed high quality diets^24^. Additionally, increased consumption of animal tissue leads to higher assimilation efficiency, increased energy storage, and higher reproductive effort in *A. pisonii*^25^. Thus, higher quality diet may reflect increased animal consumption (cannibalism, fiddler crabs, insects, etc.) and may play an important role in facilitating production of larger clutches comprised of higher quality offspring during lunar phase with the highest tidal amplitudes.

### Hypothesized breeding strategy of crabs

Varpe et al.^19^ proposed that temperate water copepods used stored capital to produce high quality offspring during the initial part of the reproductive period, and that they turned increasingly to income breeding for subsequent egg clutches produced later in the season. The results here do not demonstrate conclusively that this same pattern is followed by *A. pisonii*, but they are suggestive. The declining egg mass throughout the reproductive period suggests that capital became depleted as time went on, and that lower quality eggs with less lipid were the result. This suggests that stored capital was most useful early in the reproductive period. At the same time, the increased clutch size during new moon phases later in the breeding season, after individual egg masses had already declined, suggests an increased reliance later in the season on income breeding. Based on this reasoning, *A. pisonii* may prioritize capital breeding early in the season and income breeding later on once capital has become depleted, as proposed by Varpe et al.^19^. Additionally, this appears to reflect a tradeoff between production of smaller clutches of high quality offspring earlier in the reproductive season, and larger clutches of lower quality offspring later in the season. Building off of the foundation described by Varpe et al.^19,20,39^, we hypothesize where each breeding strategy should be most common based on the length of the reproductive and nonreproductive periods annually. Specifically, we hypothesize that capital breeding will predominate in high latitude crab species that only produce a single clutch of eggs annually. We hypothesize that income breeding will predominate in lower latitude species that have continual reproduction year round with limited nonreproductive time available to build energy stores. And we hypothesize that species at mid and lower latitudes where there is a discrete reproductive period with multiple clutches produced annually will rely on a combination of capital and income strategies, as we have shown here with *A. pisonii*. Added flexibility in financing reproduction using both capital and income strategies increases the known range of behavioral, physiological, and life history flexibility in *A. pisonii* in particular^40^ and in crabs more broadly^41,42^. This flexibility may help explain the ability of crabs to succeed under widely varying conditions, making them successful invaders around the world^43^.

Additional research is needed to test these hypotheses and to enable conclusions about the relative importance of capital and income breeding in crabs, as well as the occurrence of mixed strategies or switching between strategies, in this important group of consumers. In addition, data on foraging and energy storage outside the reproductive season are lacking for this species. Future research that describes these patterns could shed light on the use of capital breeding early in the reproductive season. Finally, it is not clear how the timing of a possible shift from capital to income breeding strategies may be influenced by year-to-year differences in the syzygy tide inequality cycle. However, for *A. pisonii*, this cycle plays an important role in foraging and energy storage strategies. Accounting for this cycle in future work will therefore help explain variation in energetics that may further clarify patterns of capital and income breeding.

## Methods

### Crab collection

*Aratus pisonii* in Florida reproduces from June to October, with few ovigerous females found in May and November and none from December to April^24^. Thus, we collected female crabs of reproductive size (11.7–23.6 mm CW, mean 18.1) from March to October 2018. This allowed us to examine reproductive effort leading up to and through most of the reproductive season.

In order to ensure the collection of ovigerous crabs (when present), we took advantage of the lunar synchronization of *A. pisonii* reproduction^21^ by collecting crabs each month 0–6 days before the full moon and/or new moon. Sampling was conducted monthly in March and April (before the new moon), and twice-monthly (before full and new moons) from May–August. Sampling effort was increased during this peak of the reproductive season in order to increase sample size and temporal coverage. Logistical and safety constraints (i.e., hurricanes) prevented sampling before the new moon in September and October, and collections were only made before full moons of these months (Table 2).

**Table 2:** Sampling sites and dates of collections from each site.

Site	Lat–Long	Sample Period 1	Sample Period 2	Sample Period 3
Pepper Park	27° 29′ 42″ N 80° 18′ 12″ W	12/03/2018 (NM, n = 23)	11/06/2018 (NM, n = 21)	08/08/2018 (NM, n = 22)
North Causeway	27° 28′ 28″ N 80° 19′ 12″ W	13/04/2018 (NM, n = 19)	26/06/2018 (FM, n = 23)	20/08/2018 (FM, n = 20)
Oslo	27° 35′ 14″ N 80° 21′ 55″ W	15/05/2018 (NM, n = 21)	11/07/2018 (NM, n = 23)	21/09/2018 (FM, n = 22)
Round Island Park	27° 33′ 33″ N 80° 19′ 53″ W	25/05/2018 (FM, n = 20)	25/07/2018 (FM, n = 22)	23/10/2018 (FM, n = 24)

Letters in parentheses following each sampling date refer to the lunar phase (NM = new moon, FM = full moon), followed by the sample size.

We collected crabs across four sites on a rotation (Table 2) to avoid having an undue impact on the population at any one location. Sites were chosen for accessibility and similarity. All four sites are tidally-dominated secondary mangrove stands with semi-diurnal tides and relatively modest tidal amplitudes (< 1 m), have muddy substrates, and are located on the Atlantic Intracoastal Waterway of the Atlantic coast of Florida between 27.42°N and 27.47°N (Table 2). The limited geographical extent of the sampling area reduced environmental difference between sites. However, to account for any differences across sites, we included collection site as a random factor in all analyses, as described below. The sample site “North Causeway” consists of a fringing mangrove stand located next to a busy boat launch in a ~ 60 m stretch between a well-traveled road and the Intracoastal Waterway. The sites “Pepper Park” and “Round Island” are both 30–35 m fringing mangrove stands that abut embankments created to separate mosquito impoundments from the Intracoastal Waterway. “Oslo” is a protected natural area located ~ 330 m from the Intracoastal Waterway. It is next to a boat launch at the end of a gravel access road, but is relatively undisturbed. Consequently, sites differed in the degree of human impact. We accounted for differences among sites statistically, as described below. During each sampling period, we collected female, mature-sized crabs by hand (see Table 2 for sample sizes). We immediately placed crabs on dry ice and kept them frozen at − 80 °C until dissection.

### Dissections

Crabs were thawed and brought to room temperature. We then measured the wet body weight, carapace width, and noted the presence of eggs attached to the pleopods (i.e., whether a crab was ovigerous). Crabs were then dissected by removing the dorsal portion of the carapace. We first noted the presence or absence of yolk deposition, indicating ongoing vitellogenesis, determined by ovary color (vitellogenic individuals of this species have orange/red ovaries, while nonvitellogenic individual have tan/white ovaries). We next extracted and measured the width of the cardiac stomach at the ventral anterior margin (i.e., the gut width). We then separated the hepatopancreas, the ovaries, the gut, the total egg clutch (if present), and the rest of the body into separate, pre-weighed aluminum boats. Each of these were dried separately for approximately 72 h at 65 °C, and then placed into a desiccator until further processing. After 24 h, we determined the dry weights of each of these components to the nearest 0.01 mg. Finally, we removed 10 individual dried eggs from each clutch and weighed each egg using a Metler Toledo XPR2U microbalance to the nearest 0.1 μg.

### Data analyses

As described above, our sampling encompassed both new moon and full moon periods. Maximum tidal heights at full and new moons vary slightly and cycle on a 14-month period (i.e. the syzygy tide inequality cycle)^38^. In the region where we conducted our sampling, the difference in maximum height of spring tides at new and full moons is on average only 0.03 m. Despite this small difference, reproductive patterns of intertidal crab species are often synchronized so that peak spawning corresponds to the highest of these two spring tide periods^36,37^. Our sampling started during the last month of the cycle where perigee (i.e., the moon is closest to the Earth in its elliptical orbit) and syzygy (i.e., sun, moon, and Earth are all aligned) occurred during the full moon, causing the full moon high tide to be higher than the new moon high tide (March, 2018). In April 2018 the two spring tides were equivalent. Starting in May 2018 and for the rest of our sampling, perigee and syzygy coincided during the new moon, and so the new moon had higher tides than the full moon. Our collections at any given site were largely conducted at either new moon or full moon (though not entirely, see Table 2). We therefore accounted for this by including moon phase as a fixed effect in our statistical analyses as described below.

#### Establishing the reproductive season

We first examined the factors that determined whether a crab was vitellogenic or not and whether it was ovigerous or not (i.e., whether or not crabs were engaged in reproduction at all) in order to describe the start and the peak of the reproductive season. We conducted two generalized linear mixed effects model (GLM) with a binomial distribution that examined whether crabs were vitellogenic or ovigerous (yes/no). As fixed predictor variables we included crab size (CW), both the linear and second order polynomial terms for Julian collection date (to look for the expected hump-shaped relationship in reproduction indicative of the beginning and end of the reproductive season), and moon phase. We also included collection site as a random effect. For both response variables, we fit a full model including all of these predictor variables, as well as all simpler models (all including the random effect of sample site) and selected the best fitting model as the model with the lowest AIC and where ΔAIC > 2.0 for all other models. When ΔAIC ≤ 2.0 for two or more models, we selected the model with the fewest terms, based on Occam’s Razor.

We next conducted a series of analyses to test the hypotheses given in Table 1. Each of these analyses uses a different response variable in the analysis, as described below. But for each of these, we fit three models: a model with only Julian sampling day as a fixed effect, a model with only moon phase (new moon or full moon) when sampling occurred as fixed effects, and model that included both of these variables together as fixed effects. We treated individual crabs as the unit of replication, and in each model we included collection site as a random effect to account for multiple samples at each site and for differences between sites. We compared these three models using AIC, and selected the best-fitting model as described above. Using this general strategy, we tested the hypotheses given in Table 1 as follows.

#### Is stored energy sufficient to finance reproduction?

We determined whether sufficient energy is stored at the start of the reproductive season to support egg production, testing whether it is possible for eggs to be produced by capital energy storage alone (#1 in Table 1). We use the mass of the ovary and the hepatopancreas combined as a proxy for the amount of energy contained in these two organs. This is possible because, based on bomb calorimetry, energetic content of the ovary and hepatopancreas each increase linearly with mass (R^2^ = 0.99 and 0.85, respectively) (Griffen, unpublished data). While the hepatopancreas is the primary site of long term energy storage, we conservatively combined it with the ovaries in this analysis to account for the possibility that some energy had already been transferred to the ovary in preparation for egg production. The first ovigerous crabs of the season were observed in May (see Results). We therefore determined the egg mass for ovigerous crabs encountered in May (i.e., the first clutch of the season) and the mass of the hepatopancreas + ovaries for crabs captured in March and April (i.e., the mass of total energy that could be devoted to egg production before any eggs were produced) and compared these with a t-test. We also compared the CW of crabs during these two sampling periods to make sure that any differences were not due to differences in the size of sampled crabs. If the mass of the hepatopancreas + ovary exceeds the mass of the first clutch of eggs, then this would suggest that enough energy is stored to produce this single clutch of eggs using capital alone. Otherwise, this suggests that egg production for even this single clutch cannot be supported by capital energy alone. This is a conservative test of this hypothesis because it uses only the first clutch of eggs rather than all clutches produced throughout the reproductive season.

#### Energy storage

We examined changes in energy storage using the mass of the hepatopancreas as a proxy to determine whether energy stores decrease throughout the reproductive season, consistent with capital breeding (#2 in Table 1). Hepatopancreas mass is expected to increase with crab size. We therefore first regressed hepatopancreas mass on crab mass and used the residual from this relationship as the response variable in our analysis. A decline in energy storage throughout the reproductive season would be consistent with capital breeding, anything else would be consistent with income breeding.

#### Egg mass and number

We examined the mass and number of eggs separately (#3 in Table 1). We examined the mass of eggs using a linear mixed effects model and treating crab ID nested within collection site as a random effect to control for repeated measures on the same crab (we measured the mass of 10 eggs for each crab). We used residual egg mass after accounting for crab CW, because egg mass increases weakly with crab size (see Results). We quantified the number of eggs produced by individual crabs by dividing the total mass of the clutch of eggs by the average mass of an egg for that crab. We then regressed egg number on crab size (CW) and used the residual clutch size from this regression as the response variable in our analysis. A decrease in egg mass or number throughout the reproductive season would be consistent with capital breeding, anything else would be consistent with income breeding, or would suggest that an excess of energy is stored prior to the reproductive season to provision a constant size and number of eggs.

#### Diet quantity and quality

We examined the amount and quality of food consumed throughout the reproductive season to determine whether energy increases proportional to reproductive effort, consistent with income breeding (#4 in Table 1). We examined the quantity of food consumed by regressing gut mass on body mass and again using the residual from this analysis as the response variable, because larger crabs are expected to eat more. This admittedly is a limited metric because it only provides a single snapshot of the amount of food consumed and does not account for digestion between consumption and collection. Additionally, it is a slight overestimate of the mass of food consumed because the gut mass included both the consumed food and the mass of the stomach tissue itself. An increase in gut mass during times of increased egg production would be consistent with income breeding.

Assessing diet composition using gut content analysis can often be difficult in crabs because of their omnivorous nature, the fact that they shred and masticate food upon consumption, and the mixing of food at different states of digestion within the cardiac stomach. Fortunately, relatively short-term changes in diet patterns can be assessed using crab gut morphology. As with other crab species^44^, the cardiac stomach (hereafter “gut”) of *A. pisonii* increases in size as diet quality decreases, presumably to enable sufficient energy/nutrient consumption on a lower quality diet^45^. Animal tissue represents a higher quality diet for *A. pisonii*^25^, and so crabs that consume more animal tissue, or other high nutrient food sources, generally have smaller guts^44^. Gut size changes over short time intervals reflecting diet changes that occur on the order of weeks^45^, and thus gut size can be used as a reliable indicator of overall changes in diet quality throughout the reproductive season in this species^24,43^. We therefore regressed gut width against carapace width and used the residual from this regression as the response variable in our analysis to assess diet quality. We included the standard set of predictor variables given above, but in addition we also used residual gut mass as a predictor variable, reasoning that guts with more food in them may stretch, thereby yielding larger gut width measurements than guts that are empty.
